# The comprehensibility and feasibility of the modified brief pain inventory and fear of pain questionnaire adapted for children and young people with cerebral palsy

**DOI:** 10.1007/s11136-025-03981-4

**Published:** 2025-04-29

**Authors:** Meredith Grace Smith, Rachel J. Gibson, Mathew Schibani, Remo N. Russo, Abirami Thirumanickam, Adrienne R. Harvey

**Affiliations:** 1 School of Allied Health Science and Practice, The University of Adelaide North Terrace, Adelaide, SA 5005 Australia; 2 Novita, Adelaide, Australia; 3 Women’s and Children’s Hospital, Adelaide, Australia; 4 Paediatric Rehabilitation Department, Women’s and Children’s Hospital, Adelaide, Australia; 5 College of Medicine and Public Health, Flinders University, Adelaide, Australia; 6 Neurodisability and Rehabilitation, Murdoch Children’s Research Institute, Melbourne, Australia; 7 Medicine, Dentistry and Health Sciences, University of Melbourne, Melbourne, Australia

**Keywords:** Measurement, Chronic pain, Cerebral palsy, Patient-reported outcome measure

## Abstract

**Purpose:**

To test the comprehensibility and feasibility of the modified brief pain inventory (mBPI) and Fear of Pain Questionnaire for Children short-form (FOPQ-C-SF) adapted for children and young people with cerebral palsy (CP), with diverse cognitive and communication abilities. Improving assessment of pain interference (mBPI) and pain-related fear (FOPQ-C-SF) in CP can enhance quality of life by increasing access to under-utilised interventions targeting pain-related physical disability and mental health.

**Methods:**

A convergent mixed methods approach was used. Twenty-two people (5–30 years) with CP completed the adapted mBPI and FOPQ-C-SF in a cognitive interview, administered by pen/paper or *TalkingMats*^®^, an evidence-based visual communication framework. Cognitive interviewing approaches were adapted to optimise participation and expression for diverse cognitive and communication abilities. Quantitative data were analysed to report tool administration times, overall completion rates and communication effectiveness. Qualitative data were analysed by content analysis to determine further changes required to the tools.

**Results:**

Median administration times were 6.2 min (IQR = 5.3–7.6) for mBPI and 4.1 min (IQR = 2.7–4.9) for FOPQ-C-SF. All completed the mBPI. Three did not complete the FOPQ-C-SF due to fatigue, challenging behaviour and parent recommendation. Ten minor changes were identified.

**Conclusion:**

The adapted mBPI and FOPQ-C-SF are likely comprehensible and feasible for children and young people with CP, including those with diverse cognitive and communication abilities. It is likely most children and young people with CP can effectively communicate responses to both tools. The adapted tools will now undergo further psychometric testing, prior to becoming freely available for clinical and research use.

**Supplementary Information:**

The online version contains supplementary material available at 10.1007/s11136-025-03981-4.

## Abstract (plain language summary)

Up to three-quarters of children and young people with cerebral palsy report chronic pain, which is much higher than those without cerebral palsy. Despite this, chronic pain is often poorly managed, with best practice pain management options being under-utilised by people with cerebral palsy. Improving how chronic pain is assessed can lead to better pain management, which can improve a person’s quality of life. Although pain questionnaires are available, many are not suitable for children and young people with cerebral palsy with different communication, cognitive and movement abilities. Previous work by our team has adapted two pain questionnaires for children and young people with cerebral palsy: The Fear of Pain Questionnaire for Children and the modified Brief Pain Inventory. The aim of this study was to test the adapted pain questionnaires in children and young people with cerebral palsy to ensure they were practical to use and were understood appropriately. To ensure this, people with cerebral palsy who also have different communication, cognitive and movement abilities were included. This study found that the adapted questionnaires are likely understood and practical to use for children and young people with cerebral palsy, including those with different abilities. The adapted questionnaires can now be further tested and then made freely available for clinical and research use. Using the adapted questionnaires in clinical practice may help to identify people who could benefit from pain treatments and connect them with supports.

**Supplementary Information:**

The online version contains supplementary material available at 10.1007/s11136-025-03981-4.

## Introduction

Children and young people with cerebral palsy (CP) experience higher rates of chronic pain than other paediatric populations, with reported prevalence up to 76% [1]. Noyek et al. (2023) identified pain intensity as the most common chronic pain domain assessed in young people with developmental disabilities [2]. However pain intensity contributes little to understanding chronic pain within the context of a person’s life. Pain should instead be assessed within a biopsychosocial framework [3]. A challenge when applying a holistic chronic pain assessment framework is that many available tools are not validated in CP, or lack suitability across the spectrum of motor, communication and cognitive abilities in the CP population [4]. Cognitive impairment can make it challenging to meet the cognitive demands required of a self-report assessment, including attention to task, memory and judgement [5]. Practically, this includes understanding the item itself, relating the item to the individual’s experience, understanding the response scale and making an evaluation of which response best represents the individual. For a child or young person with CP, this may be further compounded by the additional cognitive and motor demands of using augmentative and alternative communication (AAC), or fine motor limitations (i.e. for writing or circling the items). Despite these challenges, efforts to support individuals with diverse cognitive and communication abilities to self-report their pain should be prioritised [2]. Improved pain assessment has the potential to increase access to currently under-utilised best-practice chronic pain interventions, leading to better pain management and an overall improvement in quality of life [6].

Previous research by our team has identified two tools for potential use in CP [7]: the Modified Brief Pain Inventory (mBPI) [8], a modified version of the Brief Pain Inventory assessing pain interference in children and young people with neuromuscular disorders, and the Fear of Pain Questionnaire for Children – Short Form (FOPQ-C-SF) [9], which assesses pain-related fear and activity avoidance in children. Pain interference and pain-related fear/activity avoidance are key factors to consider when evaluating the impact of pain on quality of life [10]. Furthermore, these factors are often targets for chronic pain interventions, highlighting the importance of accurate assessment. For example, improving the assessment of pain-related fear could lead to better identification of children and young people with CP who may benefit from psychological interventions, helping them to manage their chronic pain more effectively and, ultimately, improving their participation in activities meaningful to them.

Both tools have undergone evaluation of two components of content validity (relevance and comprehensiveness) to identify modifications to improve appropriateness and accessibility for children and young people with CP, which have since been completed [7]. The modifications implemented have sought to reduce cognitive demand and improve accessibility, including by simplifying wording, using alternative administration methods and adding visual symbols. Further investigation of comprehensibility was recommended, defined as ‘ensuring tools are understood as intended by the population of interest’ [11].

Therefore, the primary aim of this study was to evaluate the comprehensibility and feasibility of the newly adapted tools in children and young people with CP, encompassing a range of cognitive, communication and motor abilities. A secondary aim was to determine if further changes were required. We aimed to answer the following research questions:

1. Are the adapted assessment tools comprehensible for children and young people with CP, with diverse cognitive, communication and motor abilities?
2. Are the adapted assessment tools feasible for use with children and young people with CP, including those who use AAC?
3. Are further changes required to improve the comprehensibility and feasibility of the tools?

## Methods

### Study design

This study employed a convergent mixed methods approach [12], integrating prospective observational (quantitative) and qualitative descriptive components [13]. Both quantitative and qualitative data were concurrently collected and analysed: the quantitative to examine administration time and effectiveness of communication interactions, and the qualitative using modified cognitive interviewing to understand children and young people’s perceptions. This approach allowed for a comprehensive analysis of the research topic, adhering to the consolidated criteria for reporting qualitative research (COREQ) [14] and guided by the COnsensus-based Standards for Measurement Instruments (COSMIN) [11]. The study was approved by the Women’s and Children’s Health Network Human Research Ethics Committee (2022/HRE00154) and guided by a lived experience advisory group, including two young adults with CP and a parent of a child with CP, which was representative of the spectrum of motor, cognitive and communication ability seen in CP. The advisory group met bimonthly throughout the project and were reimbursed for their time. They provided guidance on all aspects of the project including the research questions, participant recruitment strategies, interview engagement strategies, piloting the interview guide, reviewing the qualitative coding schema and assisting with interpretation of findings.

### Participants and setting

Children and young people were eligible for inclusion if they met the following criteria: (a) diagnosis of CP, (b) 5–30 years old, (c) able to communicate a simple response by any method and (d) experienced ‘everyday pain’, defined as ‘pain which is recurrent or chronic with acute exacerbations’ [6]. The advisory group recommended the age group inclusion criteria to 1) ensure the modifications made to the tools to enhance cognitive accessibility enabled understanding for a person with the cognitive ability of a five-year-old child; and 2) to extend the cut-off for young adulthood to 30 years, recognising this period may be extended for individuals with disability due to delayed transitions to adulthood social roles. It was expected that most participants would fall within levels I-III of the Communication Function Classification System (CFCS), meaning they would typically be able to communicate effectively with at least familiar partners, in order to meet inclusion criteria c). There were no specific exclusion criteria. Children and young people were recruited by online advertising and flyer distribution through Novita and the Women’s and Children’s Paediatric Rehabilitation Department. Maximum purposeful sampling was employed to facilitate inclusion of varied ages and abilities. Written informed consent was obtained from young adults without cognitive impairment, caregivers of children and caregivers of young adults with cognitive impairment. The ethics approved protocol contained guidelines to ensure children and young people understood the study (where possible) and/or to obtain assent.

### Sample size

COSMIN consider testing content validity in a qualitative study of seven or more participants from the population of interest as ‘very good’, and four to six participants ‘adequate’ [11]. Given the heterogenous population, we chose to target 15–20 children and young people in a larger single round, rather than multiple smaller rounds. A similar approach was used by Kramer and colleagues, when testing the comprehensibility of a functional performance patient-reported outcome measure (PROM) [15]. We also chose to target at least 4 participants in each subgroup of interest (age group, motor ability, cognitive ability and communication ability) to ensure adequate representation.

## Materials

### *TalkingMats* versions of the mBPI and FOPQ-C-SF

One key recommendation from our previous work was to consider alternative administration methods for the tools [7]. *TalkingMats Ltd*, an evidence-based visual communication social enterprise was engaged to develop an alternative for each tool. *TalkingMats* has been previously used to support understanding and improve the expression of views of people with disability [16]. An iterative process including reviewing the modifications, reaching agreement on further minor modifications and selecting visual symbols was undertaken (MGS, ARH, RJG, RNR, *TalkingMats Ltd*, advisory group). This resulted in adapted versions of the mBPI and FOPQ-C-SF, in two formats: pen/paper and *TalkingMats*. The *TalkingMats* response scale contained three symbols, with each item able to be placed either under a symbol or in between the symbols, producing a 5-point scale consistent with the paper version. Typically *TalkingMats* uses only positive wording for the response option on the left side of the mat, however we used a negative phrase to enable direct comparison between the paper and *TalkingMat* versions [16]. The pen/paper version also included identical response symbols, along with the same visual symbols for each item.

### Effectiveness framework of functional communication

The Effectiveness Framework of Functional Communication (EFFC) was used alongside cognitive interviewing to determine the young person’s understanding of the tool and ability to provide accurate responses. The EFFC was chosen as it is a valid and reliable tool assessing the effectiveness of a communication interaction and considers domains such as engagement and understanding of both the thinker and listener [16, 17].

### Data collection

Children and young people were offered a choice of interview location; home, a community site or online. Interviews were 30–60 min duration and conducted by one of two interviewers (MGS, MS) between November 2023 and January 2024. All interviews were audio-visually recorded, transcribed verbatim and able to be ceased at any time. Interviews with children were conducted with a caregiver present, with some children and young people known to MS/MGS through clinical relationships. Children and young people were randomly assigned a *TalkingMats* or paper version of the mBPI and FOPQ, unless they required a specific version for both as determined by the caregiver and interviewer (Table 1). Caregivers and support people were requested to provide communication support only, focusing on eliciting the participant’s voice. Caregivers were asked to withhold their perceptions and views on the questions until the end of the interview, with interviewers reiterating this during the interview as necessary. Interviewers recorded field notes both during, and immediately following, the interview (template in Online Resource 1). Approaches from the literature were combined to develop an interview guide (Online Resource 2) with three main stages (Table 2) [15, 18–20].

**Table 1:** Strategies implemented to facilitate participation from young people with varying cognitive and communication abilities at different stages of the project

Recruitment	Prior to the interview	During the interview	After the interview
Asking clinicians to invite young people to participate rather than relying solely on online advertising	Discussing communication methods and approaches for the young person with the caregiver/communication support person prior to the interview	Allowing a support person to be present during the interview	Allowing asynchronous participation- option to leave symbols with the young person after the interview for further consideration
Specifically inviting AAC users on the flyer	Caregivers and the interviewer were able to select the most appropriate version of the tools for testing (and able to switch to a different administration method during the interview if needed)	Visual support	
	Allowing asynchronous participation - including the option to view symbols in advance	Advising support people of the importance of eliciting the participant’s voice in the interview rather than that of the support person	
		Allowing longer interview times for AAC users	
		Video recording interviews to capture non-verbal communication	

**Table 2:** Interview guide stages – further specific information provided in online resource 2

Stage 1	Observation of the tool being completed, including asking the young person to ‘think out loud’ if possible
Stage 2	Probing questions focused on particular items, response options, symbols/pictures, examples, clarifying interpretation
Stage 3	Overall experience of the tool, supported by a visual evaluation in the form of a simple 3-point Likert scale. If applicable, parents were also engaged in this stage and asked to provide feedback on their child’s engagement with the process, understanding and preference for administration method

The interview guide was tested by the advisory group, who were asked to reflect on their experience as a young person/their own child’s experience and suggest changes. As the *TalkingMats* and pen/paper versions use identical questions, symbols and response options, the administration method was not expected to affect comprehensibility. However, it was anticipated to influence feasibility, including the participant’s engagement in the cognitive interview itself. For this reason, the advisory group recommended specifying the administration method (*TalkingMats* vs. paper) could be changed during the interview if it was not meeting the needs of the interviewee. Furthermore, interview methods were adapted to each person’s abilities, using evidence-based strategies to optimise participation and expression for those with varying cognitive abilities [21, 22] and those who used AAC [23] (Table 1).

### Data analysis

The EFFC was scored independently by two of three authors for each young person (MGS, ARH, MS). An overall score was provided for each tool completed by the young person. Following initial scoring, meetings were held to reach consensus on each item and overall score. Where consensus was unable to be reached, a third author was involved. Consensus EFFC scores were totalled for each tool and version. A score of ≥ 75% (21) was considered ‘effective’ communication as per Murphy et al. (2010) [17]. Each item on the EFFC is scored on a 0–4 scale (0 = never/none, 4 = always). For example, a score of 75% could be obtained by scoring ‘often = 3’ for each of the six domains (engagement, thinker’s understanding of the issue for discussion, listener’s understanding of thinker’s views, thinker on track, symmetry, real time and listener’s satisfaction). Quantitative interview data was descriptively analysed to report tool administration duration, overall completion rates, missing items and consensus EFFC scores.

Qualitative data, including videos, transcripts, interview notes and completed questionnaires were analysed by a two-stage content analysis (Table 3) [13, 24]. This adapted approach ensured research questions could be answered practically and specifically, while still allowing for deeper exploration [24]. All data was analysed independently by two of three authors for each young person (MS, ARH, MS). Data from children and young people using AAC devices were also analysed by an experienced speech pathologist (AT). Transcripts and quotes were offered for review. NViVO 12.0plus software (QSR International, Melbourne, Australia) was used to facilitate analysis. The final coding schema was triangulated with the authors, advisory group and EFFC scores. This was an iterative process conducted by the authors and the advisory group, resulting in the final coding framework (Table 5) and item matrix table (Online Resource 3). The item matrix table was used to identify items with unintended interpretation and track changes to each specific item in each tool (Online Resource 3). All suggested changes were reviewed by the authors and advisory group to reach consensus on the final version (Table 6, Online Resource 3).

**Table 3:** Stages of qualitative content analysis

Stage 1 - deductive	Stage 2 - inductive
1. Initial familiarisation with data • Watching video recordings • Reviewing transcripts/interview notes • Reviewing final questionnaires	1. Sub-category classification – including potential explanations for ‘unintended interpretation’ comprehensibility data
2. Coding data to pre-determined big picture coding categories related to the research questions (comprehensibility, feasibility, suggested changes, other)	2. Consensus determination for sub-category definition/description (ARH, MGS, MS)
3. Coding comprehensibility data as ‘intended’ or ‘unintended’ interpretation in relation to item (wording and symbol), instructions and recall period	4. Continued sub-category coding
	5. Review of the coding schema and all suggested changes (all authors and advisory group), including triangulation with EFFC scores to determine possible reasons for unintended interpretation and feasibility issues
	6. Iterative discussion with advisory group and research team to finalise the coding schema, item matrix table and the changes to be implemented

### Researcher characteristics and reflexivity

Content analysis recognises the researcher’s perspectives and prior experiences may influence data analysis [13]. MGS is a female PhD student and physiotherapist, and MS is a male occupational therapist, both with > 9 years’ experience working with children and young adults with CP. AT is an experienced qualitative researcher and speech pathologist working with children who use AAC. ARH, an experienced disability researcher and physiotherapist; RR, a paediatric rehabilitation medical specialist and researcher, and RJG, an experienced researcher in both quantitative and clinical studies, supervised the research. MGS, MS and ARH all underwent formal training in *TalkingMats*.

## Results

Twenty-two children and young people with CP and diverse motor, communication and cognitive abilities participated (Table 4, Online Resource 4). Qualitative data were coded to two main categories and seven subcategories (Table 5). The quantitative and qualitative results have been integrated and are presented within the two main categories of feasibility and comprehensibility. A full list of quotes is provided in Online Resource 5.

**Table 4:** Participant demographics:

Participant characteristic	% (*n*)	Mean (SD)
Age	*n* = 22	14.91 years (6.21 years) Age range: 5–29 years
5–12 years	36.4% (8)	
13–17 years	31.8% (7)	
18–30 years	31.8% (7)	
Sex		
Male	54.5% (12)	
Female	45.5% (10)	
Gross Motor Function Classification System (GMFCS) level		
I	13.6% (3)	
II	45.5% (10)	
III	22.7% (5)	
IV	13.6% (3)	
V	4.5% (1)	
Manual Ability Classification System (MACS) level		
I	31.8% (7)	
II	36.4% (8)	
III	4.5% (1)	
IV	22.7% (5)	
V	4.5% (1)	
Communication Function Classification System (CFCS) level		
I	40.9% (9)	
II	40.9% (9)	
III	13.6% (3)	
IV	4.5% (1)	
Communication ability in relation to pain		
Able to report and describe pain without any additional assistance	81.8% (18)	
Able to report and describe pain with the use of a communication device or other method	18.2% (4)	
Parent-reported cognitive impairment (CI)^		
*Likely*	50% (11)	
mild	9.1% (2)	
moderate	31.8% (7)	
unsure	4.5% (1)	
*Unlikely*	50% (11)	
*Formal cognitive assessment completed*		
No	72.7% (16)	
Yes	27.3% (6)	
School type	(*n* = 15)	
Mainstream without additional support	9.1% (2)	
Mainstream with additional support	36.4% (8)	
Special unit	22.7% (5)	
Work type	(*n* = 7)	
Higher education	4.5% (1)	
Full time employment	4.5% (1)	
Part time employment	9.1% (2)	
Supported employment	4.5% (1)	
Day options	4.5% (1)	
Other	4.5% (1)	
CP distribution		
R unilateral	36.4% (8)	
L unilateral	9.1% (2)	
Bilateral - lower limbs	31.8% (7)	
Bilateral - all four limbs	22.7% (5)	
Predominant motor type		
Spasticity	54.5% (12)	
Mixed	27.3% (6)	
Dyskinesia	18.2% (4)	

^cognitive impairment was determined by asking the young person/caregiver if a formal cognitive assessment had been undertaken. If yes, the result was recorded. If no, the caregiver was asked to suggest a level of cognitive impairment for the young person (none, mild, moderate, severe, unsure)

**Table 5:** Qualitative coding schema with definitions

Category	Subcategory	Subcodes	Description	*n*= (/22)
*Comprehensibility*			The participant’s ability to understand the questions and response options	21
	Intended interpretation		The participant interpreted the item, response options, instructions, symbols as intended by the research team	20
	Unintended interpretation		The participant did not interpret the item, response options, instructions or symbols as intended by the research team, or it was unclear if it was interpreted correctly	10
		Accuracy of response	Interpreted accuracy of the participant’s response based on observations by the interviewer, caregiver, and data analyser	4
		Additional clarification	Items or concepts which required additional explanation to be understood	7
		Observed challenges with comprehension	Observed difficulty understanding the item, tool or instructions based on observations by the interviewer, caregiver, and data analyser	10
		*Literal interpretation*	Items interpreted literally rather than more generally	2
	Visual presentation preferences		Specific preferences identified for visual presentation or changes to the visual presentation to enable understanding. This includes formatting and visual symbols	11
	Response option preferences		Specific preferences identified for the response options	6
Feasibility			The participant’s ability and willingness to complete the tool	12
	Missing items		Items not answered or unable to be answered by the participant. For example, considers a certain level of discomfort as normal and therefore does not associate pain with that activity	3
	Unreliable answers due to behaviour or distractibility		Answers to items seen as unreliable due to poor engagement, interpreted as due to behaviour, fatigue or distractibility	4
	Version preference		Preference expressed or observed for Talking Mat or Pen/paper version	12

### Comprehensibility

**Fig. 1:**
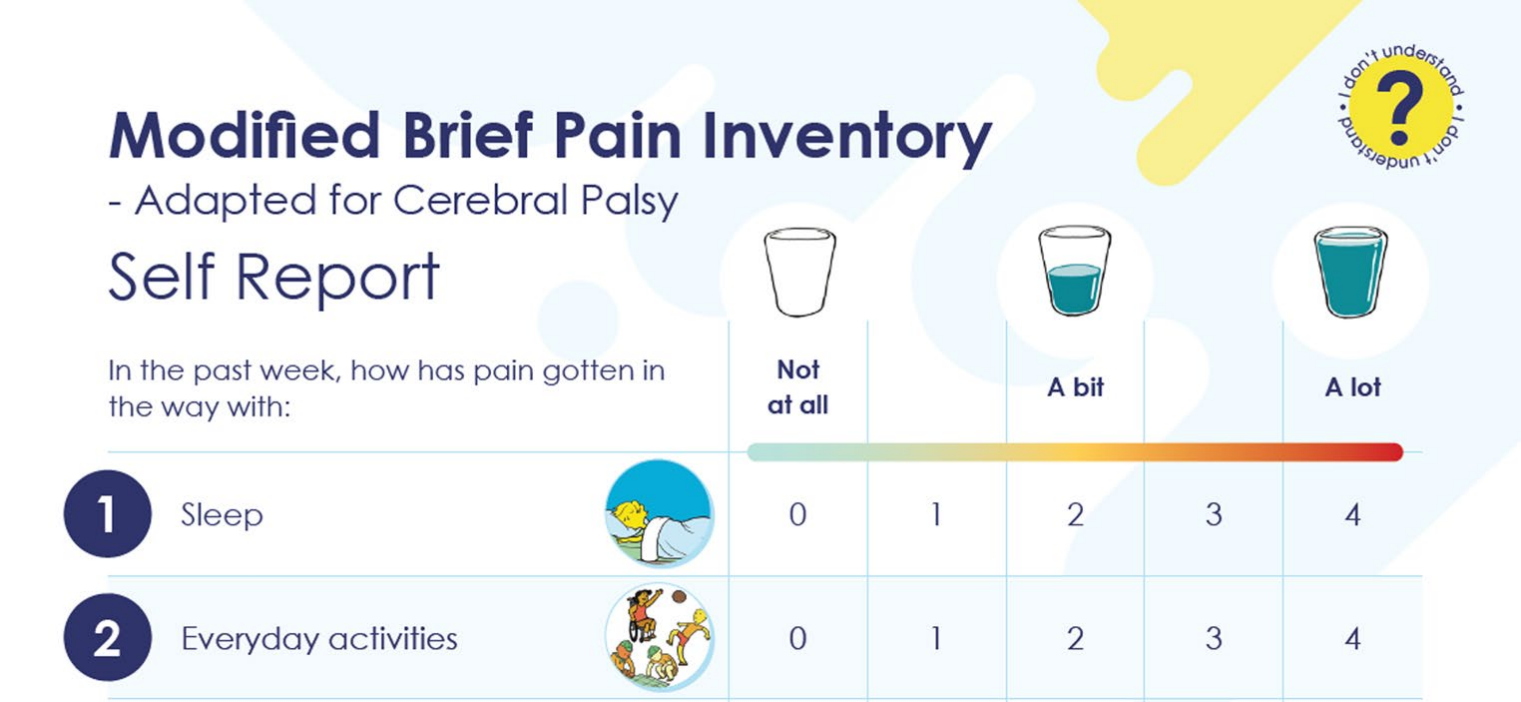
Example of the first two items and response scale of the mBPI. Images used with permission. *Abbreviations***mBPI =** modified Brief Pain Inventory

Data relating to comprehensibility were coded from twenty-one children and young people. Comprehensibility data were further coded to four sub-categories, which included intended or unintended interpretation of the tools. An item matrix table tracking all unintended interpretations of items, response options and instructions, along with the interpretation of the research team and advisory group and any changes made can be found in Online Resource 3. No interpretation issues identified were solely related to a particular administration method (*TalkingMats* vs. paper). All the interpretation issues identified were relevant to both, as they related to the item wording, item examples, response option wording or visual symbols. Consensus EFFC scores were > 21 for *n* = 17/22 (mBPI) and *n* = 15/19 (FOPQ-C-SF), indicating that most children and young people appropriately communicated responses to the tools. The qualitative data also showed that most items and response options were interpreted as intended (*n* = 20), as shown by one person’s reasoning for selecting 2 = ‘a bit’ (Fig. 1):

Just very angry sometimes when my leg starts to hurt…. just sometimes but not all the time, so I just chose the middle one because half the time it does and half the time it doesn’t.

**18 yo male, CFCS II, moderate CI, mBPI paper**

**Fig. 2:**
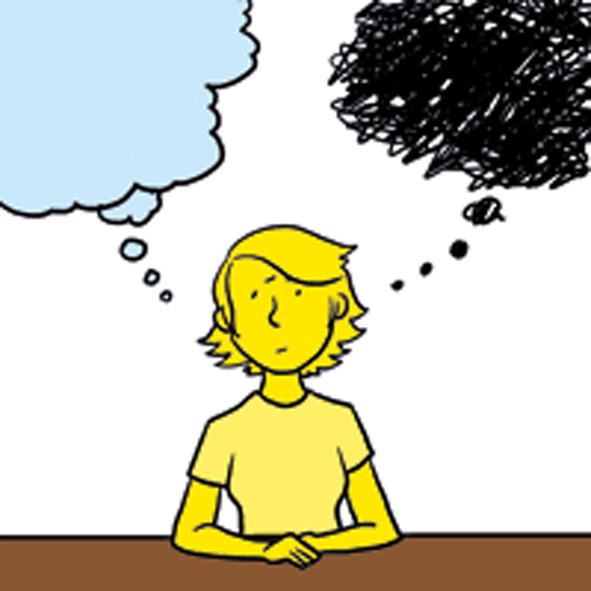
FOPQ-C-SF item 9 image (“Pain makes me think something bad will happen”). Used with permission. *Abbreviations***FOPQ-C-SF** = Fear of Pain Questionnaire for Children Short Form

The inclusion of standardised examples for items that were more general, along with the visual symbols, appeared to improve accuracy of interpretation. Children and young people demonstrated understanding of even abstract items, for example when answering ‘pain makes me think something bad will happen’ (FOPQ-C-SF item 9 – Fig. 2):

The skin might rip off and….the…. plate [in my foot] might move when someone hits it or I hit it by accident.

**9 yo female, CFCS I, no CI, FOPQ-C**, ***TalkingMats***.

### Possible reasons for unintended interpretation

Within the unintended interpretation sub-category, three sub-codes were identified as possible contributors: accuracy of response, observed challenges with comprehension and additional clarification. The five children and young people who did not reach the effectiveness threshold on the EFFC all had at least one item coded as ‘unintended interpretation’, demonstrating both reduced understanding and engagement. It was difficult to be certain of the response accuracy in cases where cognitive ability was low or behaviour prohibited engagement, for example when a young person was trying to complete the questions quickly. For one young person, it was not clear if they were making a joke or if they did not understand the item ‘how much has pain gotten in the way of school’ (Online Resource 5, quote 3 – mBPI Talking Mat). For children and young people who used AAC, parents provided suggestions to clarify the accuracy of the response (i.e. wording or symbol explanation). In relation to selecting a response option symbol for the mBPI:

I think a water glass [symbol] would work. [She] will typically look and then look away for a bit. She’ll look like she’s not engaged but what she’s doing is processing. Usually, it just takes a little while.

**Parent of 20 yo female**,** CFCS III (AAC user)**,** moderate CI**,** mBPI Talking Mat**.

Additional clarification by providing further examples was suggested for two mBPI items. Two children and young people suggested alternative examples for everyday activities (mBPI item 2), including caring for pets (Online Resource 5, quotes 1 and 11). Picture symbols were generally well received, however four children and young people, all with cognitive impairment, initially interpreted the visual symbols literally by focusing on the task itself rather than how pain interferes with it. When looking at the symbol for ‘looking after myself’ (picture of a person brushing their teeth):

*“Facilitator: What about pain getting in the way of you helping to look after yourself?*

*Participant: I brush my teeth.*

*Facilitator: You brush your teeth.*

*Participant: I was brushing my teeth in the bathroom.”*

**11 yo**,** CFCS II**,** moderate CI**,** mBPI Talking Mat**.

### Response option preferences & visual presentation preferences

Children and young people were explicitly asked to identify preferences between circles or a water glass for the mBPI response option symbols (Online Resource 2). All except one preferred the water glasses or did not have a preference. An empty glass symbol was recommended for ‘not at all’ rather than the original image of a glass with a small amount of water in it (Fig. 1).

### Feasibility

#### Completion times and missing items

**Fig. 3:**
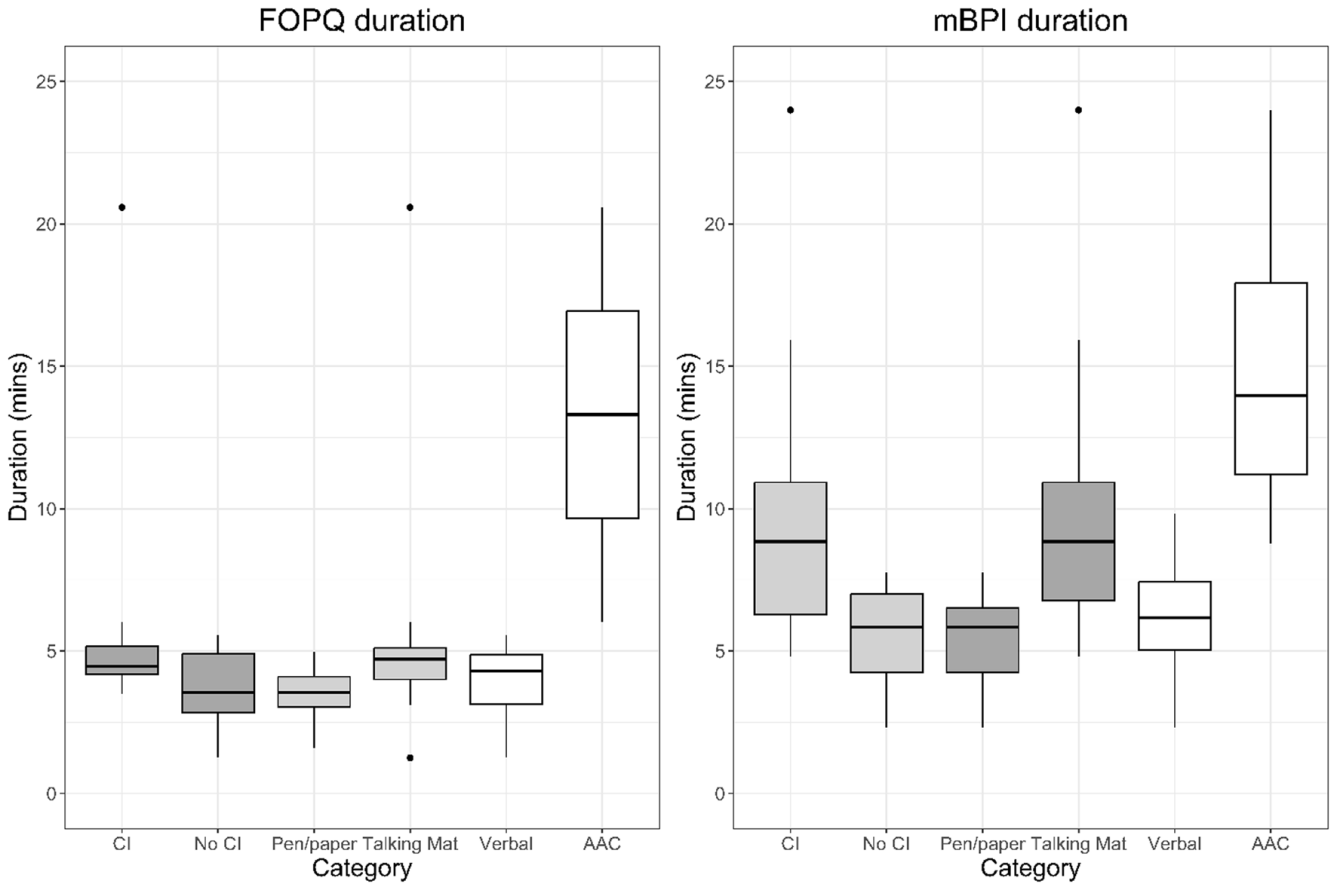
Boxplot of completion times of the adapted Fear of Pain Questionnaire Short Form (FOPQ) and Modified Brief Pain Inventory (mBPI), comparing differing communication needs, cognitive abilities and versions of the tools. **Abbreviations: AAC =** augmentative and alternative communication, **CI =** cognitive impairment, **FOPQ =** Fear of Pain Questionnaire for Children Short Form adapted for cerebral palsy, **MBPI =** modified Brief Pain Inventory adapted for cerebral palsy

Median completion times were 6.2 min (IQR = 5.3–7.6) for mBPI and 4.1 min (IQR = 2.7–4.9) for FOPQ. Completion time for AAC users and those with cognitive impairment increased by + 4.2 min (mBPI), + 9.6 min (FOPQ) and + 1.8 min (mBPI), + 1.4 min (FOPQ), respectively. Completion of *TalkingMats* versions took longer than paper (+ 2.3 min mBPI, + 1.1 min FOPQ). Table 6; Fig. 3 show the median completion times for each tool, by category of participant. Figure 3 visualises the increased time taken for AAC users, with one participant taking up to 24 min to complete the mBPI. The extended time resulted from the participant needing additional processing time for each item and requiring breaks during the interview due to fatigue. Subsequently, they did not fully complete the FOPQ-C-SF. A further two children and young people (one AAC user and one verbal communicator) did not fully complete the FOPQC-S-F due to challenging behaviour and parent recommendation. Although all children and young people completed the mBPI, seven missing items (9.8% of total data) were identified in three assessments: everyday activities (*n* = 1), school/work/day activities (*n* = 1), looking after myself (*n* = 2), having fun (*n* = 1), mobility (*n* = 1) and favourite thing (*n* = 1). Of the participants with missing items, all had reduced communication ability with unfamiliar people (CFCS level III and IV) and cognitive impairment. Identified reasons for missing items included difficulty disentangling the impact of pain and impact of disability (mBPI – mobility and looking after myself), inability to choose one favourite thing (mBPI - favourite thing), difficulty understanding abstract questions due to cognitive limitations (mBPI - having fun) and lack of relevance as the individual did not attend school, work or respite due to complex health needs (mBPI - school/work). For example, after a young person did not answer ‘looking after myself’ (mBPI item 6), the caregiver speculated this may be because it is difficult to separate the impact of disability and impact of pain as the young person has significant motor limitations:

Whereas I would say, from my perspective here, pain does affect her capacity to let us do things or participate in her own care. She really doesn’t want self-care to happen when she’s sore… she’s like ‘I want to stay in bed, leave me alone’.

**Parent of 20 yo female**,** CFCS III**,** moderate CI**,** mBPI Talking Mat**.

Another young person expressed difficulty knowing if they were answering the questions correctly, because they were ‘so used to being in pain’:

*I’m so used to being in pain but it also depends on how much you’ve pushed yourself how much physical activity you’ve done. I do actually [have] a bit of a [high] pain tolerance*,* due to all the other stuff I’ve been through. So I may not be able to give the best answers.*

**14 yo female**,** CFCS I**,** no CI**,** mBPI paper**.

For one younger child, ‘pain makes me not want to make plans’ (FOPQ item 7) was not felt to be relevant, however this was still answered by all children and young people.

*Facilitator: Pain makes me not want to make plans.*

*Participant: No. Not me.*

*Facilitator: You still like to make plans?*

*Participant: I don’t really make plans [Mum makes them]*.

**6 yo female**,** CFCS I**,** no CI**,** FOPQ-C paper**.

**Table 6:** Median duration completion times by tool and category (in minutes)

Category	mBPI median (IQR)	FOPQ median (IQR)
Overall	6.2 (5.3–7.6)	4.1 (2.7–4.9)
Verbal	6.2 (4.8–7.4)	3.7 (2.5–4.6)
AAC user	10.4 (9.6–11.2)	13.3 (9.7–16.9)
CI	7.6 (6.1–9.1)	4.5 (4.2–5.2)
No CI	5.8 (4.2-7)	3.1 (1.8–4.8)
Pen/paper	5.8 (4.2–6.5)	3.5 (2.3–3.9)
Talking Mat	8.1 (6.1–9.1)	4.6 (3.1-5)

*Abbreviations***AAC =** augmentative and alternative communication, **CI =** cognitive impairment, **FOPQ =** Fear of Pain Questionnaire Children Short Form adapted for cerebral palsy, **mBPI =** modified Brief Pain Inventory adapted for cerebral palsy

#### Version preference

Accessing picture symbols was challenging for one young person who also had a visual impairment, reporting he ‘just listened’ to the questions as he could not see the pictures:

*Facilitator: The other thing that I wanted to know is what you thought of the pictures. Because I don’t know if you were using them*,* or whether you were just listening.*

*Parent: More to say*,* categories. Actions. Sensory actions… We’re wondering*,* all the pictures that were on the cards*,* whether you could see them*,* whether you were looking at the pictures*,* or more to say*,* go to categories. Turn the page. Something’s wrong. Turn the page. You couldn’t see them. Listening*,* could not see*.

*Participant: [Nods*,* indicating yes]*

*Parent: So*,* with the pictures… you couldn’t see them?*

*Participant: [Nods*,* indicating yes].*

*Facilitator: You couldn’t see them… So*,* you were listening?*

*Parent: Categories*,* action words. Sensory actions. Were you listening to the descriptions? Is that how you were answering the questions*,* using your listening?*

*Participant: [Nods*,* indicating yes].*

**11 yo male**,** CFCS III (AAC user)**,** no CI**,** mBPI/FOPQ Talking Mat**.

**Fig. 4:**
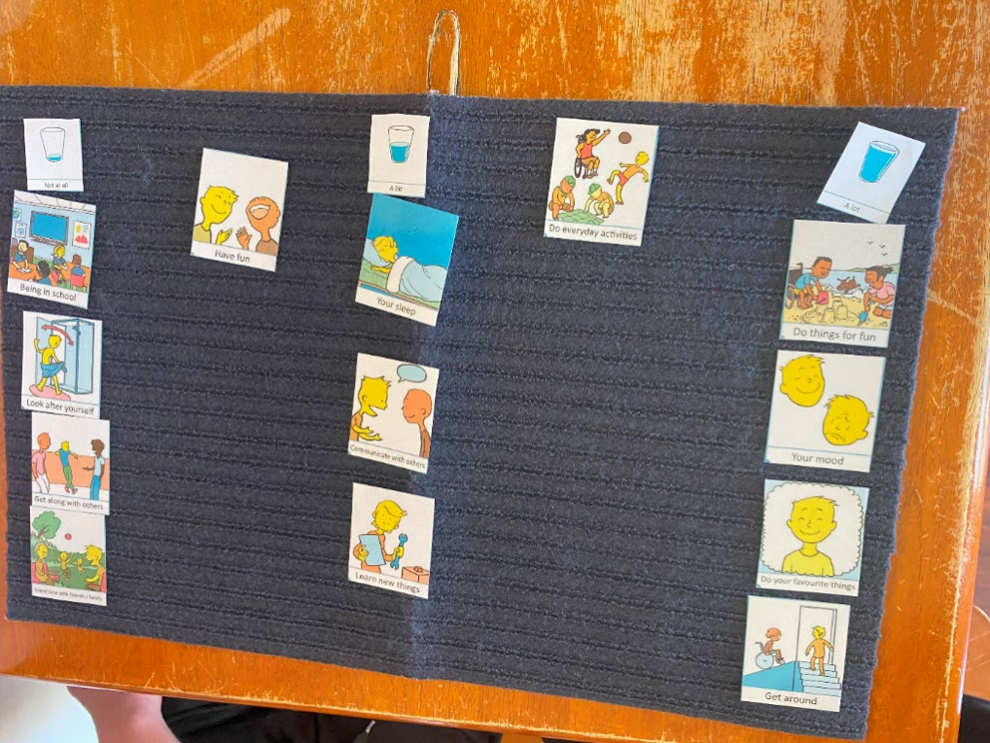
Example of a completed Talking Mat for the mBPI. Used with permission. *Abbreviations*: **mBPI =** modified Brief Pain Inventory

The paper version was preferred for older children and those without cognitive impairment, however all were still able engage with the *TalkingMats* version. *TalkingMats* (Fig. 4) was preferred by younger children, AAC users and those with cognitive impairment (quote 24, Online Resource 5).

### Further suggested changes

Twenty-two suggested changes were identified during inductive coding (Online Resource 6). Implemented changes are listed in Table 7, with item progression from original to final tracked in Online Resource 3.

**Table 7:** Changes implemented after data review by author team and consumer advisory group

Implemented changes	Related category	Tool	Version
Remove ‘day activities’ from school/work/day activities as this caused confusion with mBPI item 2 ‘everyday activities’. Instead include ‘day options/respite’ as an example in the administration guide	Comprehensibility	mBPI	Both Talking Mat and paper
Alternate background row colour	Comprehensibility	mBPI & FOPQ-C	Paper
Make font slightly larger	Feasibility	mBPI & FOPQ-C	Paper
Put a line either side of the middle response option on the paper version	Comprehensibility	mBPI & FOPQ-C	Paper
Simplify introduction for the FOPQ-C-SF	Comprehensibility	FOPQ-C	Talking Mat & paper
Use an empty glass image rather than slightly full for the ‘not at all’ response option for the mBPI	Comprehensibility	mBPI	Talking Mat & paper
Use the water glass images over the circle images for the mBPI	Comprehensibility	mBPI	Talking Mat & paper
Have FOPQ talking mat response options on the same side as the paper version (i.e. ‘this is not me on the left’ and ‘this is me on the right’ – Fig. 5)	Comprehensibility	FOPQ-C	Talking Mat
Initially remove the middle symbol from the Talking Mat if an individual struggles to choose from three options due to cognitive ability	Feasibility	mBPI, FOPQ-C	Talking Mat
Add ‘or helping to look after myself’ to mBPI item 6 (looking after myself) for those who have limited independence	Comprehensibility	mBPI	Talking Mat & paper

**Fig. 5:**
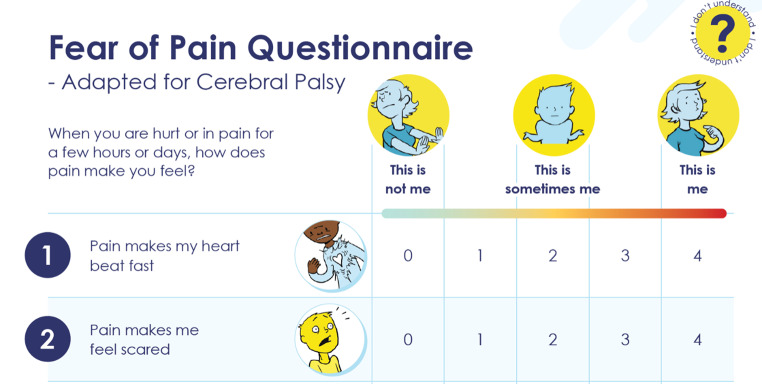
Example of the first two items and response scale of the FOPQ-C-SF. Images used with permission. *Abbreviations*: **FOPQ-C-SF** = Fear of Pain Questionnaire for Children Short Form

## Discussion

This study examined the comprehensibility and feasibility of adapted versions of the mBPI and FOPQ-C-SF in children and young people with CP and varying motor, communication and cognitive abilities. The study found the adapted tools are both comprehensible and feasible for most children and young people with CP, including AAC users and those with mild-moderate cognitive impairment. Ten minor adjustments will be implemented prior to wider testing, and as missing items made up less than 10% of the data, no items will be deleted [25]. From a clinical perspective, this means that children and young people with CP can and should be encouraged to provide self-reported pain information using these newly adapted tools. Regular use of these adapted tools in clinical practice may provide the first step in identifying self-reported pain interference and pain-related fear in children and young people with CP, subsequently facilitating access to best-practice chronic pain interventions, such as positive pain coping strategies, which can improve quality of life [6, 26].

When assessing comprehensibility, approximately three quarters of children and young people demonstrated ‘effective communication’ on the EFFC while completing the mBPI and FOPQ-C-SF. The ability to engage with the assessment was also dependent on how concrete or abstract the concepts were. The FOPQ-C-SF had lower EFFC scores and fewer full completions than the mBPI, likely because almost all the items are abstract in nature. Literal interpretation of the item symbols was a common issue for young people with a cognitive impairment in this study, and was similarly noted in a previous study adapting a PROM for people with intellectual disability [15]. This was managed by providing additional examples to help the participant relate the symbol to the intended activity. For the participants in our study who did not meet the ‘effectiveness’ threshold, the assessment still provided a degree of valuable self-reported information that was otherwise unknown. This demonstrates the importance of exploring a young person’s own pain perspectives as a right, regardless of age or cognitive ability [5, 21]. It is therefore recommended that self-report tools are used as first priority, and if the young person is unable to complete the whole tool or there is concern about self-report accuracy, a caregiver can provide an additional proxy report. This ensures proxy-report is not the default, as parents may find it difficult to estimate the impact of their child’s pain [27]. In this study, the interplay between behaviour, fatigue, concept abstractness and familiarity with the interviewer likely impacted EFFC scores, subsequent ‘effective communication’ rating and overall comprehensibility. In clinical practice, strategies can be implemented to improve ‘effective communication’ when using the tools, such as being in a familiar environment with someone known to the young person.

Two of the three people with missing items were communicating using AAC and were unable to provide reasons for not responding to these items. In this case, caregivers suggested reasons for the missing items, which included difficulty disentangling the impact of pain from the other impacts of disability. For people with significant functional limitations, cognitive impairment and/or other medical conditions, the challenge of isolating the impact of pain was similar to findings by Rinde et al. 2023 [27], who explored the theme ‘my child’s pain is just one piece of a complex jigsaw puzzle’. Some people in the present study also had difficulty identifying the impact of pain because pain is a usual part of life, consistent with other literature investigating pain in children and CP [27]. Caregivers were helpful in these circumstances as they were able to suggest alternative phrases the young person might typically use instead of pain, such as ‘think about when your leg is stiff’.

The feasibility of completing the assessments was highly dependent on the administration method used. While it was originally planned to ask all participants to complete both administration methods of each tool (i.e. *TalkingMats* and paper version of the mBPI, and *TalkingMats* and paper version of the FOPQ-C), the advisory group recommended against this due to participant burden. Where possible, all participants completed one tool in the *TalkingMats* version and the other in the paper version to allow comparison between the administration methods (i.e. mBPI *TalkingMats* and FOPQ-C paper). The paper versions of the tools can be used with the support of a freely available administration guide, however *TalkingMats Ltd* requires practitioners to complete training prior to use in clinical practice. The training is low cost, accessible online and self-paced. As a result, it is unlikely to pose a significant barrier to implementation in clinical settings. The training also has broader applicability for clinicians working with people with communication disability and for people with cognitive impairment (with or without speech). *TalkingMats* was the preferred version for AAC users; however, it is still important to be flexible for individual needs. For one young person, a suggestion was made to use high-contrast symbols like those in their Pragmatic Organisation of Dynamic Display (PODD) to meet their sensory requirement. This need for flexibility was identified by parents of AAC users in our previous study [7] and is unsurprising given the high level of customisation required when developing and prescribing AAC systems [28, 29].Completion times increased for children and young people with cognitive impairment and communication access needs, with the mBPI taking up to 24 min and FOPQ up to 21 min for one AAC user. A key accessibility feature for those who use AAC is allowing them adequate time to engage [23]. Clinicians should consider how the young person can best self-report when completing the assessment and employ strategies such as completing the tool during the appointment with a clinician. This will also enable richer discussion around pain experience and management, goal setting and shared decision making. Furthermore, providing the assessment questions ahead of time can enhance communication rate for AAC users with CP, while also addressing cognitive load and associated fatigue issues [23]. The motor impairments in CP increase the complexity around operational competencies for using the AAC system, making the rate of communication slower and more fatiguing. Clinicians should reflect on their individual clinical setting and prioritise self-reporting of pain, particularly for those who need more time to respond. In time-limited clinical settings, one option is for the young person to complete the assessment with a family member and send it ahead of time to the healthcare team. However, it is essential to allocate time during the appointment to review the completed assessment and discuss its implications. It may also be useful to complete the adapted tools within broader assessments, such as surveillance services like CP-UP in Sweden [30]. 

Adapting pain assessments for those who use AAC, younger age groups and those with cognitive impairment did not change their usability or comprehensibility more broadly. This is consistent with the concept of universal design, demonstrating how improved accessibility for those with specific needs also improves usability for those without [5]. Children and young people without cognitive or communication impairment reported that the simplified language and response scales, improved formatting and images made the tools quicker and easier to use. While the adaptations have enabled at least a degree of self-report for those with mild-moderate cognitive impairment, we did not have any children and young people with severe cognitive impairment in the study, even though they were not excluded. Our observations during the study suggest it is unlikely a young person with severe cognitive impairment would have the capacity to complete the adapted assessments, although this needs to be determined. Observational tools, such as the Paediatric Pain Profile [31] may be most useful in these cases. A strength of this study was including AAC users, younger children and those with cognitive impairment, groups which are often excluded in research due to a perception that they are unable to self-report [32]. There is a natural flow-on effect from exclusion in research to exclusion in clinical practice, with most regularly used PROMs requiring verbal or written communication, and cognitive abilities of > 8 years of age [33]. Our study showed the adapted pain assessment tools are suitable for individuals as young as five years, those with mild-moderate cognitive impairment, and diverse motor and communication abilities. The broader age range also allowed comparison between general preferences for administration method, with younger children generally preferring the *TalkingMats* version and older participants generally preferring pen/paper.

While previous studies have adapted or investigated PROMs for people with intellectual disability [15, 34], we are not aware of any studies that have considered adaptations for a heterogenous group like CP, which includes people with diverse cognitive, communication and motor abilities. Participant diversity was important in identifying further refinements to the tools (Table 7). For example, the inclusion of ‘helping to look after myself’ to mBPI item 6 improves inclusivity for people with limited independence. Furthermore, strategies to reduce visual demand - such as alternating the background row colour and increasing the font size - were identified by participants with visual impairments. Similar to our study, other research has successfully improved comprehensibility by simplifying response options and adding visual symbols for younger children and people with intellectual disability [15, 35]. A unique feature of our adaptation process was the use of *TalkingMats* as a PROM administration method rather than solely a communication tool. Other research with children with intellectual disability has used *TalkingMats* alongside other arts based participatory methods, such as body mapping and photo voice, to identify key constructs of interest for patient-reported experience measures [34].

Although the study participants were skewed towards higher levels of functioning (i.e. GMFCS/MACS levels I-III), there were still sufficient participants in the lower functioning groups to be confident that saturation was reached. COSMIN consider a sample size of 4–6 participants as adequate [11], and the study included four participants in GMFCS levels IV/V and 6 participants in MACS levels IV/V. Furthermore, even from a highly heterogenous group, there were few changes to be made to the items, wording, response options and administration (Online Resource 6). Given there were so few changes to be made from such a diverse group and considering the sample size both overall and for each subgroup of participants meets the COSMIN recommendations for ‘very good’ or ‘adequate’, we are confident the tool is appropriate for use across the diversity of ability.

The study had several limitations. It is usual for multiple smaller rounds of pilot testing to be completed with changes made to the tool between rounds [25]. Given the heterogeneity of the CP population, we felt it more appropriate to initially capture the perspectives of a larger group, rather than making changes from a smaller group and potentially needing to undo those changes [7]. We had planned to complete additional pilot testing if wording changes were identified [18]. However, the only change implemented was the removal/addition of one word, and as such it was not deemed necessary to re-test.

It is possible that researcher bias influenced the analysis of observed body language/non-verbal cues, however this was minimised by involving at least two researchers at each analysis stage, all with experience working with people with CP and an experienced speech pathologist to specifically analyse AAC users. A further limitation was potential bias from having a communication support person and/or parents present during the interviews. To minimise this, interviewers spoke with the support person prior to the interview about focusing on the young person’s perspective and voice. For AAC users, the support person can also be considered a benefit given their greater familiarity with the person’s communication cues. A final limitation was possible fatigue experienced by the children and young people after completing two tools in one session. This may have impacted engagement with the second tool, however this was managed by allowing breaks.

### Future directions

The mBPI and FOPQ-C-SF adapted for children and young people with CP will now undergo further psychometric testing prior to being made freely available for use in clinical practice and research. It is possible these tools could be digitised in future, potentially decreasing administration times and allowing greater customisation. It is important to consider how a digitised version could be used alongside an AAC device, and how it would be sustainably maintained and freely accessed.

## Conclusion

The adapted mBPI and FOPQ-C-SF are likely comprehensible and feasible for use in children and young people with CP, including those with diverse cognitive and communication abilities. Tool adaptations such as alternative administration methods, visual symbols and wording simplification helped improve self-report participation for people with CP. Minor changes have been identified through the pilot testing process and will be implemented prior to further psychometric testing. Prioritising the inclusion of people with diverse cognitive and communication abilities is both feasible and critical in developing inclusive and accessible assessment tools.

## Electronic supplementary material

Below is the link to the electronic supplementary material.

Supplementary Material 1

Supplementary Material 2

Supplementary Material 3

Supplementary Material 4

Supplementary Material 5

Supplementary Material 6
