# Supplementary material for: The comprehensibility and feasibility of the modified brief pain inventory and fear of pain questionnaire adapted for children and young people with cerebral palsy
Source: Qual Life Res. 2025 Apr 29;34(8):2377–92. doi: 10.1007/s11136-025-03981-4 (PMC12274258; doi:10.1007/s11136-025-03981-4)
Supplement: Supplementary file 1 — Supplementary Material 1 [file 11136_2025_3981_MOESM1_ESM.docx]

**Interviewer name:**  **Date:**

| **Before interview:** | |
| --- | --- |
| **Participant name** |  |
| **Participant age** |  |
| **Location of interview** (home/community/online) |  |
| **Predominant motor type** (spasticity/dyskinesia/mixed) |  |
| **Has the participant experienced ongoing pain** (i.e. pain lasting longer than 3 months or longer than the expected time to heal) |  |
| **GMFCS level** |  |
| **CFCS level** (Link here: [CFCS level](http://cfcs.us/wp-content/uploads/2018/11/CFCS_English_CP.pdf)) |  |
| **MACS level** |  |
| **Child: School type** (mainstream, mainstream with education plan/additional support, special class, special unit, special school) |  |
| **Adult: Work/day activities** (independent employment, supported employment, day options, other) |  |
| **Communication device used (yes/no)**  If yes, please provide more details |  |
| **Cognitive impairment likely? (yes/no)**  **If yes, mild, moderate, severe** |  |
| **Other comments/helpful information** |  |

Check that video recording of meeting is on

Consent form signed

**Modified Brief Pain Inventory**

Picture supported scale

Talking mats – hard copy

Talking mats – digital

| **Particular questions/items/pictures participant has trouble answering or understanding**  *How did you know the participant was having difficulty?*  *Did you use ‘how much is pain changing XXXX’ or ‘how much is pain getting in the way of XXX’?* |  |
| --- | --- |
| **Observed moments of confusion/difficulty** |  |
| **Number of times participant indicated “I don’t know” or “I don’t understand”** |  |
| **Other observations** |  |

Photo of questionnaire/talking mat taken

**Fear of Pain Questionnaire for Children- Short Form**

Picture supported scale

Talking mats – hard copy

Talking mats – digital

| **Particular questions/items participant has trouble answering**  *How did you know the participant was having difficulty?* |  |
| --- | --- |
| **Observed moments of confusion/difficulty** |  |
| **Number of times participant indicated “I don’t know” or “I don’t understand”** |  |
| **Other observations** |  |

Photo of questionnaire/talking mat taken

| **After interview – from video footage – two assessors during data analysis** | | | | | | |
| --- | --- | --- | --- | --- | --- | --- |
| **Rate the participants understanding of the three circles**  Abstract questions/items of the tools are identifiable on the talking mats symbols | | | | | 1. **Consistently understood** 2. **Sometimes understood** 3. **Consistently misunderstood** | |
| 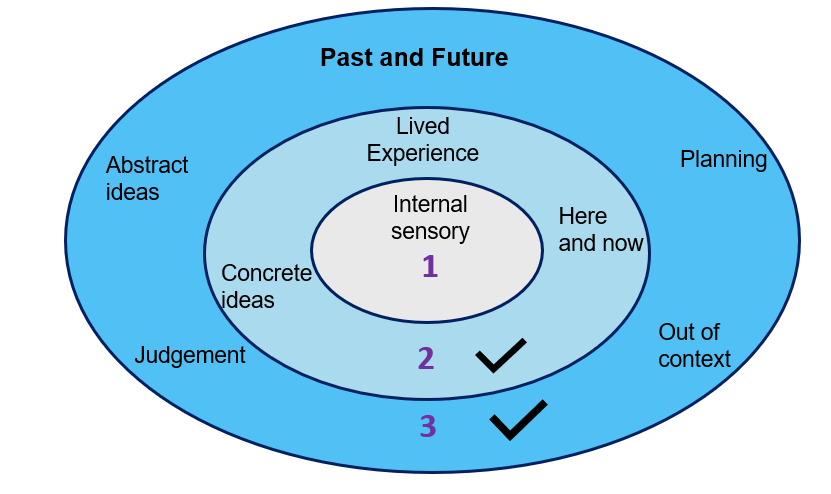 | | | **Circle 1** (internal/sensory) | |  | |
|  |  |  | **Circle 2** (Here and now, lived experience, concrete ideas, known vocabulary, on task, sorting, objects, single words) | |  | |
|  |  |  | **Circle 3** (Past and future, abstract ideas, planning, out of context, judgement) | |  | |
| **Effectiveness framework of functional communication - mBPI**  *To be completed during data analysis from video footage – 2 assessors* | | | | | | |
| Indicators | 4  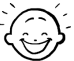  Always | 3  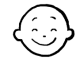  Often | | 2  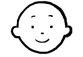  50:50 | 1  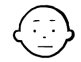  Occasionally | 0  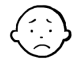  Never/none |
| **Engagement**  *the social closeness that is established in the interaction and maintained through rapport and joint attention* |  |  | |  |  |  |
| **Thinker’s understanding of issue for discussion**  *based on verbal and non-verbal responses* |  |  | |  |  |  |
| **Listener’s understanding of thinker’s views**  *shown by non-verbal and verbal responses of listener* |  |  | |  |  |  |
| **Thinker - On track**  *the relevance of the thinker’s verbal and non-verbal responses to the topic being discussed* |  |  | |  |  |  |
| **Symmetry**  *the sense of equilibrium and balance that creates shared control in the interaction* |  |  | |  |  |  |
| **Real time**  *the extent to which the interaction happens within a typical time frame* |  |  | |  |  |  |
| **Listener’s Satisfaction** *the perspective of the listener about how easy it is to support and maintain the interaction* |  |  | |  |  |  |
| **Total score: /28**  *A total score of 75% (21) or more represents effective communication.* | **Comments:** | | | | | |

| **Effectiveness framework of functional communication – FOPQ-C-SF**  *To be completed during data analysis from video footage – 2 assessors* | | | | | |
| --- | --- | --- | --- | --- | --- |
| Indicators | 4  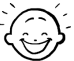  Always | 3  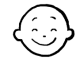  Often | 2  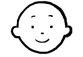  50:50 | 1  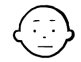  Occasionally | 0  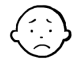  Never/none |
| **Engagement**  *the social closeness that is established in the interaction and maintained through rapport and joint attention* |  |  |  |  |  |
| **Thinker’s understanding of issue for discussion**  *based on verbal and non-verbal responses* |  |  |  |  |  |
| **Listener’s understanding of thinker’s views**  *shown by non-verbal and verbal responses of listener* |  |  |  |  |  |
| **Thinker - On track**  *the relevance of the thinker’s verbal and non-verbal responses to the topic being discussed* |  |  |  |  |  |
| **Symmetry**  *the sense of equilibrium and balance that creates shared control in the interaction* |  |  |  |  |  |
| **Real time**  *the extent to which the interaction happens within a typical time frame* |  |  |  |  |  |
| **Listener’s Satisfaction** *the perspective of the listener about how easy it is to support and maintain the interaction* |  |  |  |  |  |
| **Total score: /28**  *A total score of 75% (21) or more represents effective communication.* | **Comments:** | | | | |
