# Supplementary material for: The comprehensibility and feasibility of the modified brief pain inventory and fear of pain questionnaire adapted for children and young people with cerebral palsy
Source: Qual Life Res. 2025 Apr 29;34(8):2377–92. doi: 10.1007/s11136-025-03981-4 (PMC12274258; doi:10.1007/s11136-025-03981-4)
Supplement: Supplementary file 2 — Supplementary Material 2 [file 11136_2025_3981_MOESM2_ESM.docx]

**Tailoring Chronic Pain Assessment to Children and Young People with Cerebral Palsy**

**1. INFORMATION ABOUT THE ASSESSMENTS**

1. **Modified Brief Pain Inventory**

What aspects of pain does it assess?

This questionnaire assesses **how pain interferes with function**, including the impact of pain on emotional wellbeing. This tool has been used in children, adolescents and adults with chronic pain and disability. It covers the following pain domains:

- Impact of pain on emotional functioning
- Impact of pain on physical ability
- Impact of pain on participation
- Impact of pain on quality of life
- Impact of pain on sleep

How to complete it?

- Written questionnaire, spoken questionnaire, picture supported scale, Talking Mats version
- Proxy report and self-report versions are available

When would it be used?

- It would most likely be used as a ‘quick check’ for all people with cerebral palsy, at least yearly. It would be used to see if pain was an issue, and if so to start a conversation about how it can be better managed.

1. **Fear of Pain Questionnaire for Children**

What does it assess?

This questionnaire assesses **pain related fear** in children, adolescents and young adults with chronic pain. It covers the following pain domains:

- Impact on emotional wellbeing
- Impact of pain on physical ability
- Impact of pain on participation

How to complete it?

- Written questionnaire, spoken questionnaire, picture supported scale, Talking Mats version
- Proxy report and self-report versions available (n.b. we have not modified the parent report version)

When would it be used?

- It would most likely be used after a screening assessment had already been completed. If the screening assessment showed that pain was impacting day to day life, then it might be used to see if the **fear of pain** was also impacting day to day life.

**2. CONDUCTING INTERVIEWS**

*Each participant will complete modified versions of both the modified Brief Pain Inventory (mBPI) and the Fear of Pain Questionnaire for children – short form (FOPQ-C-SF). Please select the version you think is most appropriate for the participant:*

1. *Picture supported scale (one item/page or all items on one page)*
2. *Talking mats framework –* [*link here*](https://www.talkingmats.com/flipbook) *for overview of talking mats if needed*

*If you feel that the participant is capable of completing both versions above (i.e. could complete the picture supported scale and talking mats version), then please select the picture supported scale for one of the tools and the talking mats framework for the other (i.e. picture supported scale mBPI followed by talking mats framework for the FOPQ-C-SF) (as per the randomisation schedule).*

*Please discuss with the parent/support person if you need assistance deciding. It is also fine to start with one version and swap if it is not meeting the needs of the participant*

Talking mats – different versions of images

Make sure you have the appropriate group of images for the age of the participant (child/adolescent/young adult)

For the mBPI, there are two different top scale images – try and test out both options across participants (i.e. participant 1 uses top scale 1, participant 2 uses top scale 2).

*Pie chart top scale*: *Water glass top scale*:


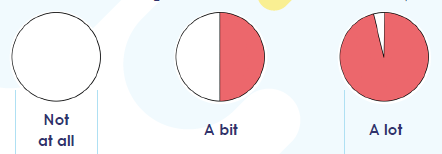

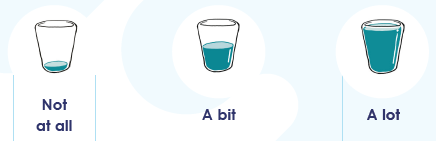


**Before starting the interview**

Set up as needed for the participant’s method of communication (i.e. set up switches, ensure communication device is ready to use)

Set up the video recording on the laptop/ipad/phone – *ensure you can see the participant’s face/upper body and the talking mats board/screen*

If using the picture supported scale, the participant can complete it independently, with the parent/support person or with you (interviewer) assisting. For talking mats version, you (interviewer) will complete with the child

**Conducting the interview:**

1. Choose which tool to complete first – *it doesn’t really matter, but the modified brief pain inventory is less abstract than the fear of pain questionnaire (so may be easier for participants)*
   1. For talking mats, must ask concrete items first/abstract items last
2. If possible, ask the participant to ‘think out loud’ while they are answering the questionnaire
   1. “Can you tell me what you are thinking while you are answering each question”
   2. Could ask an open ended question about the item– ‘Tell me a bit more about your pain when you sleep’? to clarify if they are responding on the scale consistently with what they describe (i.e. if they rate positively on the scale about sleep, are their open ended responses about sleep positive also)
      1. Example – ‘pain makes me think something bad will happen’ – “what would something bad be for you?”
3. Questions after completing the tool (dependent on communication method – can also ask parents for their opinion on these questions)

*Can choose to use closed or open questions depending on how the participant is able to provide information-* Please use the **post interview visual evaluation** to ask the following questions:

- 1. Closed questions
     1. Were there questions/items/pictures that you did not understand? Which question? *Can use the pictures here to clarify*
     2. Did the pictures help you to understand? Which pictures?
     3. Did the examples help you to understand? Which examples?
     4. Were there words that were hard to understand?
     5. Which pictures helped you understand the best?
  2. Open questions
     1. What did item XXXX mean to you?
     2. What did picture XXXX mean to you?
     3. What does XXX weeks (recall period) mean to you? Tell me about your pain over the last XXX weeks
     4. How did you feel about the time it took?
     5. How did you feel about understanding the questions?
     6. How did you feel about the pictures?
     7. How did you feel about the examples given?
     8. What do you think of the answer options?
     9. Why did you choose answer A over answer B?
     10. Do you have any ideas on how we could make this easier to use?
     11. Were there any questions that you found really hard to answer? Why?
     12. How did you feel about using the paper scale?
     13. How did you feel about using the mat version?
     14. How did you feel about being listened to?
  3. For parents/support person
     1. How would you go helping your child/the participant complete this without a health professional to help?
     2. Would an administration guide be helpful? If so, what mode/method would work best (i.e. short video, A4 page with instructions, etc.)
     3. What did you think of the questionnaire/assessment tool?
        1. What did you like/not like about the questionnaire?
     4. Which version did you like best?
     5. How effective were the different aspects of the tool at helping your child to answer accurately?

1. Repeat process with **the second questionnaire**

**Role of the support person**

A support person can be present for any interview. The support person will be briefed by the chief investigator prior to the interview to ensure that the participant’s views are prioritised. The support person’s role may include:

1. Assist in selecting the appropriate version of the assessment (talking mat vs pen/paper) for the participant
2. If a participant requires additional assistance to understand a question, the examples provided in the administration guides should be used first. If further description is required, the support person may help to describe the item. The interviewer is to ensure that the description provided by the support person is consistent with the meaning of the item
3. Assist the interviewer in understanding the participant’s communication cues


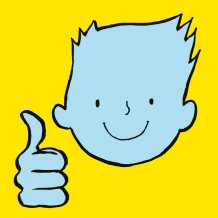

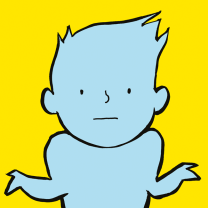




| 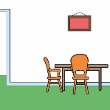How did you feel about the pictures? |  |  |  |
| --- | --- | --- | --- |
| 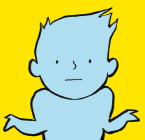 How did you feel about the answer choices? |  |  |  |
| 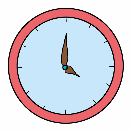How did you feel about the time it took? |  |  |  |
| 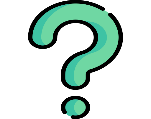 How did you feel about the questions? |  |  |  |
| 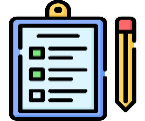How did you feel about the paper scale? |  |  |  |
| 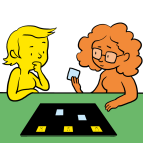 How did you feel about using the talking mat? |  |  |  |
| 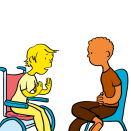How did you feel about being listened to? |  |  |  |

**Not happy**

**Okay**

**Happy**
