# Supplementary material for: The comprehensibility and feasibility of the modified brief pain inventory and fear of pain questionnaire adapted for children and young people with cerebral palsy
Source: Qual Life Res. 2025 Apr 29;34(8):2377–92. doi: 10.1007/s11136-025-03981-4 (PMC12274258; doi:10.1007/s11136-025-03981-4)
Supplement: Supplementary file 3 — Supplementary Material 3 [file 11136_2025_3981_MOESM3_ESM.docx]

**Supplementary material: Cognitive interviewing interpretation of items and changes made**

| **mBPI original item** | **Interpretations different to those of the research team** | **Comments – research team and advisory group** | | **Changes made to the item** | **Final item** |  |
| --- | --- | --- | --- | --- | --- | --- |
| Introduction lines: *In the past week, how has pain gotten in the way with:* | #27: didn’t understand ‘pain’, so changed to ‘in the past week, how has your legs hurting gotten in the way of…’  #30: used ‘did pain stop you doing XXXX…. Yes/No’ as the participant could only answer binary responses using communication devices | May need to use an example specific to the individual if they have trouble understanding pain  May need to be adaptable for participants who use AAC | | No | Introduction lines: *In the past week, how has pain gotten in the way with:* |  |
| Recall period: *In the last week* | #24: Couldn’t remember if the pain had been in the last week or longer  #36: wanted to answer based on the time of day – said this can be different over the week | Some concrete thinkers had difficulty considering the past week.  Advise test administrators to ask the participant to consider their last week in general | | No | Recall period: *In the last week* |  |
| Sleep | #24: answer dependent on if she had her period or not  #1: “is this about getting to sleep or staying asleep?’ | Required prompting to consider the recall period (past week)  Participant to consider sleep in general | | No | Sleep |  |
| Everyday activities | #35: requested examples for everyday activity – used examples in the guide  #26: “Just fill up the dog bucket and empty the dishwasher”  #14: “spend time with pets” | Examples provided in the administration guide can be used to support understanding  Add ‘caring for pets’ to the examples list | | No | Everyday activities |  |
| Mood | #30: Mum advised that participant’s PODD uses ‘feelings’ rather than ‘mood’ | May need to adjust wording slightly for AAC users to be consistent with the wording they are used to. No other participants had difficulty with this phrase | | No | Mood |  |
| **School/work/day activities** | #1: wanted to add ‘sport’ as the participant was an elite athlete  #33: Did not present this item as parent said it was not relevant (participant had been at home due to medical complexities for the past week)  “Is day activities the same as everyday activities?” | This participant was able to understand this question related to the activity that most filled her day, and answer this question accordingly.  Day activities was used to encompass day options programs/respite. This was confusing because of item 2, ‘everyday activities’. This has been reworded | | **Yes** | **School/work**  **(includes respite, day options, study)** |  |
| Things I do for fun | Nil |  | | No | Things I do for fun |  |
| **Looking after myself** | #22: answered specifically about brushing teeth  #18: answered specifically about having a shower  #33: parent reworded this to ‘helping to look after’ as the participant has limited independence in activities of daily living | Some concrete thinkers need prompting to consider more broadly than the exact picture being displayed  Consideration for those who have limited independence in activities of daily living – would be helpful to use the term ‘helping to look after myself’. However for those who do have independence, the original wording is more appropriate | | **Yes** | **Looking after myself (or helping to look after myself)** |  |
| Learning new things | Nil |  | | No | Learning new things |  |
| Getting along with others | Nil |  | | No | Getting along with others |  |
| Communicating with others | Nil |  | | No | Communicating with others |  |
| Having fun | Nil |  | | No | Having fun |  |
| Spending time with friends and family | Nil |  | | No | Spending time with friends and family |  |
| Getting around | #22: not answered – parent reported this is because there are factors other than pain contributing to the ability to do these things  #18: ‘its hard getting up the stairs’ | Answered based on the literal image (which had stairs on it). Some concrete thinkers need help to consider the item more broadly than just the picture displayed | | No | Getting around |  |
| My favourite thing to do: | #35: ‘I don’t have a favourite thing’  #27: could not pick a favourite thing | This item is optional | | No | My favourite thing to do: |  |
| **FOPQ-C-SF original item** | | **Interpretations** | | **Comments – research team and advisory group** | **Changes made** | **Final item** |
| **Introduction lines: *These questions ask about how you look at pain when you hurt or are in pain for a few hours or days. Please read each statement carefully. Indicate how much you agree or disagree with each statement.*** | | #35: ‘introduction lines are too wordy – should be deleted and just have ‘pain makes me feel’ instead | |  | **Yes** | **Introduction lines: *When you are hurt or in pain for***  ***a few hours or days, how does***  ***pain make you feel?*** |
| Pain makes my heart beat fast | | #8: Had not ever noticed heart beating fast because of pain | |  | No | Pain makes my heart beat fast |
| Pain makes me feel scared | | #22: the picture looks like he is yawning | | Only one participant interpreted this as yawning. Some participants may need further explanation of the item in conjunction with the picture | No | Pain makes me feel scared |
| Pain makes me worry | | Nil | |  | No | Pain makes me worry |
| Pain makes me not want to go to things | | #11: Interpreted the picture as “he is upset because there is no cake at the cake shop” | | Some participants may need further explanation of the item in conjunction with the picture. This is particularly important for those who are unable to read the words independently | No | Pain makes me not want to go to things |
| Pain makes me want to stop any activity when my body hurts | | #14: advised he did not understand the question. Needed the item reworded specifically to him (his parent provided an example situation) | | Sometimes need to provide an example specific to the individual to help them to understand the question – this can be provided by parents | No | Pain makes me want to stop any activity when my body hurts |
| Pain makes me not want to go to school/work because it makes my pain worse | | Nil | |  | No | Pain makes me not want to go to school/work because it makes my pain worse |
| Pain makes me not want to make plans | | #25: “I don’t make really plans. Mum makes plans” | | Participant was still able to answer this question even though they felt that their mother made most their plans for them | No | Pain makes me not want to make plans |
| Pain makes me want to put things off | | Nil | |  | No | Pain makes me want to put things off |
| Pain makes me thing something bad will happen | | Nil | |  | No | Pain makes me thing something bad will happen |
| Pain means I can’t join in because I think my body will hurt | | #14: advised he did not understand the question. Needed the item reworded specifically to him (his parent  provided an example situation)  #18: “These [questions] are hard” | | Sometimes need to provide an example specific to the individual to help them to understand the question – this can be provided by parents | No | Pain means I can’t join in because I think my body will hurt |
