# Supplementary material for: The comprehensibility and feasibility of the modified brief pain inventory and fear of pain questionnaire adapted for children and young people with cerebral palsy
Source: Qual Life Res. 2025 Apr 29;34(8):2377–92. doi: 10.1007/s11136-025-03981-4 (PMC12274258; doi:10.1007/s11136-025-03981-4)
Supplement: Supplementary file 4 — Supplementary Material 4 [file 11136_2025_3981_MOESM4_ESM.docx]

| Participant characteristic | FOPQ-C (Talking Mats)  n=16* | FOPQ-C  (Paper)  n=5* | mBPI  (Talking Mats)  n=11 | mBPI (paper)  n=11 |
| --- | --- | --- | --- | --- |
| Age (years)  Mean (SD) | 13.9  (6.15 years)  Age range: 5-26 | 18.8  (5.8 years)  Age range 15-29 | 12.9  (4.5 years)  Age range: 5-20 | 16.9  (7.2 years)  Age range: 6-29 |
| Sex | n= | n= | n= | n= |
| Male | 7 | 4 | 8 | 4 |
| Female | 9 | 1 | 3 | 7 |
| Gross Motor Function Classification System (GMFCS) level |  |  |  |  |
| I | 2 | 1 |  | 3 |
| II | 7 | 3 | 6 | 4 |
| III | 4 |  | 2 | 3 |
| IV | 3 |  | 3 |  |
| V | 0 | 1 | 0 | 1 |
| Manual Ability Classification System (MACS) level |  |  |  |  |
| I | 7 |  | 2 | 5 |
| II | 5 | 3 | 5 | 3 |
| III | 2 | 2 | 2 | 3 |
| IV | 1 |  | 1 |  |
| V | 1 |  | 1 |  |
| Communication Function Classification System (CFCS) level |  |  |  |  |
| I | 8 | 1 | 1 | 8 |
| II | 5 | 4 | 6 | 3 |
| III | 3 |  | 3 |  |
| IV |  |  | 1 |  |
| Communication ability in relation to pain |  |  |  |  |
| Able to report and describe pain without any additional assistance | 12 | 5 | 7 | 11 |
| Able to report and describe pain with the use of a communication device or other method | 4 |  | 4 | 0 |
| Cognitive impairment (CI)^ |  |  |  |  |
| Likely | 7 | 3 | 10 | 1 |
| mild | 2 |  | 2 | 0 |
| moderate | 4 | 3 | 7 | 1 |
| unsure | 1 |  | 1 | 0 |
| Unlikely | 9 | 2 | 1 | 10 |
| CP distribution |  |  |  |  |
| R unilateral | 5 | 3 | 3 | 5 |
| L unilateral | 1 | 1 | 0 | 2 |
| Bilateral - lower limbs | 5 | 1 | 3 | 3 |
| Bilateral - all four limbs | 5 |  | 5 | 0 |
| Predominant motor type |  |  |  |  |
| Spasticity | 9 | 3 | 5 | 7 |
| Mixed | 3 | 2 | 3 | 3 |
| Dyskinesia | 4 |  | 3 | 1 |

*1 participant did not attempt the FOPQ-C on parent recommendation
