# Supplementary material for: The comprehensibility and feasibility of the modified brief pain inventory and fear of pain questionnaire adapted for children and young people with cerebral palsy
Source: Qual Life Res. 2025 Apr 29;34(8):2377–92. doi: 10.1007/s11136-025-03981-4 (PMC12274258; doi:10.1007/s11136-025-03981-4)
Supplement: Supplementary file 5 — Supplementary Material 5 [file 11136_2025_3981_MOESM5_ESM.docx]

| **Supplementary material 5: direct quotes and their category mapping** | | | | | | |
| --- | --- | --- | --- | --- | --- | --- |
|  | **QUOTE** | **PARTICIPANT** | **CATEGORY** | **SUBCATEGORIES** | **Talking Mat vs Paper** | **Specific tool & item** |
| 1 | Facilitator: What sort of chores and things do you have to do at home?  Participant: Just fill up the dog bucket and empty the dishwasher | 14 yo female, no CI, CFCS I | Comprehensibility | Additional clarification – everyday activities | Paper | mBPI  2. everyday activities |
| 2 | Facilitator: So, what do you think about doing it this way [Talking Mat]?  Participant: It’s all right, I suppose it’s an option for people that can’t do physical writing.  Facilitator: Yeah, did you have a preference yourself? Do you prefer doing pen and paper or did you like doing them like this?  Participant: I prefer pen and paper.  Facilitator: Yep and what do you prefer about that?  Participant: Probably just easier for me to do it, probably more convenient for me | 14 yo female, no CI, CFCS I | Feasibility | Version preference | Talking Mat | N/A |
| 3 | Facilitator: Yeah? All right. How much has pain gotten in the way of you being at school?  Participant: I was being at school. I was had to do computer work.  Facilitator: Did you?  Participant: Yep.  Facilitator: Did pain get in the way of that at all?  Participant: Yeah. Not.  Facilitator: Not really, So do you want to put it where you think it goes? Is it not at all, a bit, or a lot?  Participant: A lot.  Facilitator: You think it’s a lot. But you just told me that it’s not at all.  Participant: You tell me.  Facilitator: Are you tricking us?  Participant: No.  Facilitator: Is it not at all?  Participant: Not at all.  Facilitator: Not at all. All right. | 11 yo male, moderate CI, CFCS II | Comprehensibility | Accuracy of response | Talking Mat | mBPI  4. school/work/day activities |
| 4 | Facilitator: What about pain getting in the way of you helping to look after yourself?  Participant: I brush my teeth. | 11 yo male, moderate CI, CFCS II | Comprehensibility | Visual presentation preference  Accuracy of response - *Literal interpretation* | Talking Mat | mBPI  6. looking after myself |
| 5 | Facilitator: Do you like putting the pictures onto the mat?  Participant: Yeah.  Facilitator: You do like doing that?  Participant: Yeah.  Facilitator: Was it easy to understand how to put the pictures on the mat?  Participant: Yeah | 11 yo male, moderate CI, CFCS II | Feasibility | Version preference | Talking Mat | N/A |
| 6 | Parent: I think a water glass symbol would work [for the mBPI]. Caitlin will typically look and then look away for a bit [unclear]. She'll look like she's not engaged but what she's doing is processing. Usually, it just takes a little while. Have a little think | 20 yo female, moderate CI, CFCS III, AAC user | Comprehensibility  Feasibility | Difficulty understanding  Accuracy of response - *Parent involvement to clarify accuracy of communication response* | Talking Mat | mBPI  Response options |
| 7 | Parent: Whereas I would say, from my perspective here, pain does affect her capacity to let us do things or participate in her own care. She really doesn't want self-care to happen when she's sore, [unclear] I want to stay in bed, leave me alone… | 20 yo female, moderate CI, CFCS III, AAC user | Feasibility | Disentangling impact of disability and impact of pain | Talking Mat | mBPI  2. Looking after myself |
| 8 | Facilitator: Yep. What about, how much has pain got in the way of your mood? A bit?  Participant: Yeah.  Facilitator: How does it make you feel?  Participant: Just very angry sometimes when my leg starts to hurt and then everything, but – or my leg starts to hurt or everything and just sometimes but not all the time, so I just chose the middle one because half the time it does and half the time it doesn’t. | 16 yo male, moderate CI, CFCS II | Comprehensibility | Intended interpretation | Paper | mBPI  3. Mood |
| 9 | Facilitator: What about, pain makes me worry  Participant: Again, actually I’m going to change what I’ve done, I’m going to change it to something [unclear]…  Facilitator: And why did you chose that one instead?  Participant: Because if I chose the middle one again, it’s going to be [unclear] change it round again so that’s why I chose, that’s why I chose, because I chose the two beginning ones, middle, middle, so I’m just trying this one because I know this one was a little bit more hard than the others…  Facilitator: Yeah, yep, so it makes you a bit more worried than the other one?  Participant: Yes. | 16 yo male, moderate CI, CFCS II | Comprehensibility | Intended interpretation | Paper | FOPQ  3. pain makes me worry |
| 10 | Facilitator: Yeah, which one did you like better, did you have one…  Participant: The pencil better than just grabbing the laminating little note things and just putting it there.  Facilitator: What did you like about the pencil one?  Participant: So I get to show you how neat my writing is [unclear] last time, because the last time you saw my writing it was not the best.  Facilitator: That’s true, it would have been a long time ago that I’ve seen… | 16 yo male, moderate CI, CFCS II | Feasibility | Version preference | Both | N/A |
| 11 | Facilitator: What sort of things would you do for your everyday activities, say at home?  Participant: Spend time with the pets. | 18 yo male, moderate CI, CFCS II | Comprehensibility | Additional clarification – everyday activities | Paper | mBPI  2. everyday activities |
| 12 | Parent: It [pain] gets in the way when we’re trying to garden doesn’t it?  Participant: Yeah, but that was from seizures. | 18 yo male, moderate CI, CFCS II | Feasibility | Disentangling impact of pain and impact of disability | Paper | mBPI  2. everyday activities |
| 13 | Facilitator: How much does pain get in the way of how you look after yourself? What's he doing there?  Participant: He's having a shower. | 11 yo female, mild CI, CFCS II | Comprehensibility | Accuracy of response - *Intended interpretation*  Accuracy of response - *Literal interpretation* | Talking Mat | mBPI  6. looking after myself |
| 14 | Facilitator: All right, how does pain get in the way of how you get around?  Participant: Hard, up…  Facilitator: You're up the stairs? Yeah, you're the one on the stairs, I reckon.  Participant: Me on the stairs. | 11 yo female, mild CI, CFCS II | Comprehensibility | Accuracy of response - *Intended interpretation*  Accuracy of response - *Literal interpretation* | Talking Mat | mBPI  12. getting around |
| 15 | Facilitator: What about, how much does pain get in the way of your mood, so how you’re feeling.  Participant: A lot, lot.  Facilitator: A lot.  Parent: You get angry don’t you.  Participant: A lot, lot.  Facilitator: A lot, here.  Participant: No, here.  Facilitator: Here, on the other side? Bigger than a lot, the biggest.  Participant: A lot. | 7 yo male, mild CI, CFCS II | Feasibility | Response option preferences - Option for more than ‘a lot’ | Talking Mat | mBPI  3. mood |
| 16 | Facilitator: Which one did you like better? Did you like doing the pen and paper or did you like doing the pictures on the mat?  Participant: The pen and paper.  Facilitator: Yeah? Why did…  Participant: It made me feel happy.  Facilitator: Yeah? Why did you prefer the pen and paper?  Participant: Because the mat one was too easy, I think. | 18 yo female, moderate CI, CFCS II | Feasibility | Version preference | Both | N/A |
| 17 | Parent: What do you think it might say on the page if you were reading this book.  Participant: [No birthday].  Parent: Pardon? No birthday. I think that's a really good answer.  Facilitator: That's perfect  Parent: Why do you reckon no birthday?  Participant: [Unclear].  Parent: Because he's annoyed in his little chair? Why do you think he might be annoyed? You're doing fantastic. Why do you reckon he's annoyed?  Participant: Because there's no cake.  Parent: Because there’s no cake. It does look like there's no cake. That's a great answer. So you think he's sitting there upset.  Participant: There's no cake in the cake shop. | 16 yo male, moderate CI, CFCS II | Feasibility | Visual presentation preferences  Accuracy of response - *Literal interpretation* | Paper/Talking Mat | FOPQ  4. pain makes me not want to go to things |
| 18 | Facilitator: Pain makes me not want to make plans?  Participant: No. Not me.  Facilitator: You still like to make plans?  Participant: I don’t really make plans.  Facilitator: Maybe mum makes plans for you | 6 yo female, no CI, CFCS I | Feasibility | Missing items | Paper | FOPQ  7. pain makes me not want to make plans |
| 19 | Participant: [Just because] I can tell not at all barely any water, and then a bit which is half a cup, sometimes, sometimes not and a full that’s a lot...  Facilitator: Which one do you like better? Do you like the glass with water, or do you like these circles?  Participant: The glass of water | 6 yo female, no CI, CFCS I | Comprehensibility | Visual presentation preferences | Paper | mBPI  Response options |
| 20 | Parent: No. I think it was interesting hearing from [the child’s] perspective because I think, particularly around mood and feelings about pain, I would have answered differently. When I see her reactions to pain, I would have had different answers, so that’s nice to know that you’re not - when you’re not feeling pain it’s not weighing on you a lot. | 6 yo female, no CI, CFCS I | Feasibility |  | N/A | Both mBPI and FOPQ |
| 21 | Facilitator: In terms of the pictures that have kind of been added; are there any that you kind of think, don’t really match with the question?  Participant: No. I think they’re pretty well – yeah, they’re pretty well on target | 29 yo male, no CI, CFCS I | Comprehensibility | Visual presentation preferences | Paper | Both |
| 22 | Facilitator: Yeah, fair enough. Fair enough. All right. Pain makes me think something bad will happen.  Participant: Kind of, yeah.  Facilitator: Yeah. Do you have any ideas? What sort of bad things would you think might happen if you had pain?  Participant: The skin might rip off and it's just going to be - and the thingy. What do you call that thingy on my foot, mum?  Parent: You just got a plate at the moment.  Participant: The plate might move when someone hits it or I hit it by accident. | 9 yo female, no CI, CFCS I | Comprehensibility | Accuracy of response - *Intended interpretation* | Talking Mat | FOPQ  9. pain makes me think something bad will happen |
| 23 | Facilitator: The other thing that I wanted to know is what you thought of the pictures. Because I don’t know if you were using them, or whether you were just listening.  Parent: More to say, categories. Actions. Sensory actions… We’re wondering, all the pictures that were on the cards, whether you could see them, whether you were looking at the pictures, or more to say, go to categories. Turn the page. Something’s wrong. Turn the page. You couldn’t see them. Listening, could not see  Participant: [Nods, indicating yes]  Parent: So, with the pictures… you couldn’t see them?  Participant: [Nods, indicating yes].  Facilitator: You couldn’t see them… So, you were listening?  Parent: Categories, action words. Sensory actions. Were you listening to the descriptions? Is that how you were answering the questions, using your listening?  Participant: [Nods, indicating yes]. | 11 yo male, likely CI, CFCS III, AAC user | Feasibility  Comprehensibility | Visual presentation preference | Talking Mat | N/A |
| 24 | Parent: Do you have an ‘I think it’s…’ word about the activity we just did with the talking mat?  Participant: [Nods, indicating yes]  Parent: Categories. Turn the page. An opinions word about the talking mat activity. Little words. Okay, good, funny, strange, silly, crazy, just kidding, teasing. Smart, clever, lucky, confusing? Like it words.  Participant: [Nods, indicating yes]  Parent: Awesome  Participant: [Nods, indicating yes] | 11 yo male, likely CI, CFCS III, AAC user | Feasibility | Version preference - Talking Mat | Talking Mat | N/A |
| 25 | Participant: Again, actually I’m going to change what I’ve done, I’m going to change it to something [else]  Facilitator: And why did you choose that one instead?  Participant: Because if I chose the middle one again, it’s going to be [the same as the previous]…. So I’m just trying this one because I know this one was a little bit more hard than the other [previous one]  Facilitator: Yeah, yep, so it makes you a bit more worried than the other one?  Participant: Yes | 16 yo male, CFCS II, moderate cognitive impairment | Comprehensibility | Accuracy of response - Intended interpretation | Paper | FOPQ  3. Pain makes me worry |
| 26 | I’m so used to being in pain but it also depends on how much you’ve pushed yourself, how much physical activity you’ve done. I do have actually a bit of [high] pain tolerance, due to all the other stuff I’ve been through. So I may not be able to give the best answers. | 14 yo female, CFCS I, no cognitive impairment | Feasibility | Missing items – *disentangling the impact of pain from the impact of disability*  Additional clarification - *Disentangling the impact of pain from the impact of disability* | Paper | N/A |

CODEBOOK – PILOT TESTING MODIFIED VERSIONS OF THE FOPQ-C and mBPI for CP

| Category name | Description |
| --- | --- |
| **Comprehensibility** | The participant’s ability to understand the questions and response options |
| Accuracy of response | Interpreted accuracy of the participant's response based on observations by the interviewer, caregiver, and data analyser |
| Additional clarification | Items or concepts which required additional explanation to be understood |
| Observed challenges with comprehension | Observed difficulty understanding the item, tool or instructions based on observations by the interviewer, caregiver, and data analyser |
| *Literal interpretation* | Items interpreted literally rather than more generally |
| Visual presentation preferences | Specific preferences identified for visual presentation or changes to the visual presentation to enable understanding. This includes formatting and visual symbols |
| Response option preferences | Specific preferences identified for the response options |
| **Feasibility** | The participant’s ability and willingness to complete the tool |
| Missing items | Items not answered or unable to be answered by the participant. For example, considers a certain level of discomfort as normal and therefore does not associate pain with that activity |
| Unreliable answers due to behaviour or distractibility | Answers to items seen as unreliable due to poor engagement, interpreted as due to behaviour, fatigue or distractibility |
| Version preference | Preference expressed or observed for Talking Mat or Pen/paper version |
