# Supplementary material for: The comprehensibility and feasibility of the modified brief pain inventory and fear of pain questionnaire adapted for children and young people with cerebral palsy
Source: Qual Life Res. 2025 Apr 29;34(8):2377–92. doi: 10.1007/s11136-025-03981-4 (PMC12274258; doi:10.1007/s11136-025-03981-4)
Supplement: Supplementary file 6 — Supplementary Material 6 [file 11136_2025_3981_MOESM6_ESM.docx]

|  | **No. participants suggesting the change** | | **Change implemented (yes/no)** | **Reasoning** |
| --- | --- | --- | --- | --- |
|  | **Comprehensibility** | **Feasibility** |  |  |
| **Additional clarification** |  |  |  |  |
| Additional option for school/work/day activities of ‘sport’ | 1 |  | No | Only identified by one young person who was an elite athlete. This young person was able to interpret ‘work’ as ‘sport’ for her |
| Everyday activities – need to use examples, some confusion between ‘day activities’ in school/work/day activities and ‘everyday activities. Consider removing ‘day activities’ and leaving as school/work | 2 |  | Yes | Remove ‘day activities’ and instead add an * to indicate that this includes respite |
| Impact of time of day on pain | 1 |  | No | Only identified by one person, information relating to this can be gathered in separate subjective assessment |
| Needed to be more specific than just pain – i.e. use language like ‘when your leg hurts….’ | 1 |  | Yes | Add to the administration guide that the wording can be altered to be specific to the individual if needed i.e. ‘how much has *your leg aching* gotten in the way of school’? |
| Sleep – clarify if this is getting to sleep, staying asleep, etc. | 2 |  | No | The advisory group felt that asking about these specific features of sleep would reduce feasibility for people with cognitive impairment/AAC users. This information should instead be gathered in subjective assessment |
| **Visual presentation preferences** |  |  |  |  |
| Alternating background row colour | 2 |  | Yes | Easier to know which item you are answering, less likely to answer along the wrong line |
| Make font slightly larger |  | 1 | No | Unable to make font larger without spreading over two pages |
| Make numbers bold | 1 |  | No | One person only suggested, graphic designer advised against this as it would make the assessment harder to read |
| Pictures on pen paper version were too small for someone with visual impairment (all on one page version) | 1 |  | No | One item per page version is already available and can be used for visual impairment (has larger images) |
| Put a line either side of middle response option | 1 |  | Yes | Easy to implement, no negatives |
| Simplify introduction for FOPQ | 1 |  | Yes | Introduction was simplified to match the mBPI |
| Consider high contrast pictures for visual impairment | 1 | 1 | No | We were unable to feasibly implement this ourselves, however recognise that this could be done by individuals using the tool in future and we are supportive of this |
| Empty glass for ‘not at all’ instead of a little water | 4 |  | Yes | Makes sense to use an image that fits with the wording ‘not at all’ |
| Faces instead of water glasses for mBPI response option pictures | 1 |  | No | One participant only suggested this, all other participants were happy with the water glasses |
| Consider an alternative image for the ‘learning new things picture’ | 1 |  | No | One participant only suggested this, all other participants were happy with the images |
| Consider simplifying the ‘pain means I can't join in picture’ | 1 |  | No | One participant only suggested this, all other participants were happy with the images |
| Preference for water glass over circles | 4 |  | Yes | Use the water glass version as it was most preferred |
| **Response option preferences** |  |  |  |  |
| Option for more than 'a lot' | 1 |  | No | One participant only, all other participants were happy to select from the response options |
| FOPQ Talking Mat - ‘this is not me’ on left side of mat and ‘this is me on right side’ (consistent with paper version) | 3 |  | Yes | Important for ease of scoring |
| Remove the middle symbol from the Talking Mat (changing to essentially yes/no) for children with lower cognitive abilities | 1 | 1 | Yes | This is already an option within the general Talking Mats framework. If this middle symbol is removed, this will need to be recorded on the assessment |
| Those with good cognitive function sometimes wanted in between response options | 3 |  | No | People with good cognitive function recognised that adding additional in between responses would make the tool less accessible to those with some form of cognitive impairment. It is recommended that clinicians gather this information in subjective assessment instead, where relevant |
| Those with good cognitive function wanted definitions for numbers 1 and 3 | 1 |  | No | People with good cognitive function recognised that adding additional definitions for the in between responses would make the tool less accessible to those with some form of cognitive impairment. |
